# Insights on variant analysis *in silico* tools for pathogenicity prediction

**DOI:** 10.3389/fgene.2022.1010327

**Published:** 2022-11-29

**Authors:** Felipe Antonio de Oliveira Garcia, Edilene Santos de Andrade, Edenir Inez Palmero

**Affiliations:** ^1^ Molecular Oncology Research Center—Barretos Cancer Hospital, Barretos, Brazil; ^2^ National Institute of Cancer, Rio de Janeiro, Brazil

**Keywords:** *in silico*, pathogenicity prediction, bioinformatics, variant classification, next generating sequencing

## Abstract

Molecular biology is currently a fast-advancing science. Sequencing techniques are getting cheaper, but the interpretation of genetic variants requires expertise and computational power, therefore is still a challenge. Next-generation sequencing releases thousands of variants and to classify them, researchers propose protocols with several parameters. Here we present a review of several *in silico* pathogenicity prediction tools involved in the variant prioritization/classification process used by some international protocols for variant analysis and studies evaluating their efficiency.

## Background

With advances in molecular biology and the increasing affordability of its techniques, biological parameters, new organisms and pathogens, and genetic diseases can be studied through the sequencing of genetic material. The large quantities of data produced with these methods require high expertise and computational power to process, identify and classify genetic variants (a new term for mutation) that may yield scientifically relevant information.

Genomic studies allowed us to uncover information and understand the molecular mechanisms of our biology and several genetic diseases. From the sequencing of small sequences to disease-related gene panels and today to whole exome/genome sequencing we are able (in some cases) to track the origin of that disease, allowing the employment of targeted therapy, thus, deeply impacting the clinical decisions of the tested patient or, in case of inherited disease, the family (Walsh et al., 2011; Nakagawa and Fujita, 2018; Felicio et al., 2021).

These studies allowed the creation of several databases and beyond, like The Cancer Genome Atlas (TCGA), ClinVar (Landrum et al., 2018), UniProt (UniProt, 2019) and The Catalogue Of Somatic Mutations In Cancer (COSMIC) (Tate et al., 2019) and others, which provide us the curated data of the molecular alterations related to diseases and serve as a deposit for new studies. Another important source of information is GnomAD (Karczewski et al., 2020), a database containing 125,748 exome sequences and 76,156 whole genome sequences. All these databases are major contributors to past and new studies and support variant classification (Richards et al., 2015; Li et al., 2017).

**FIGURE 1:**
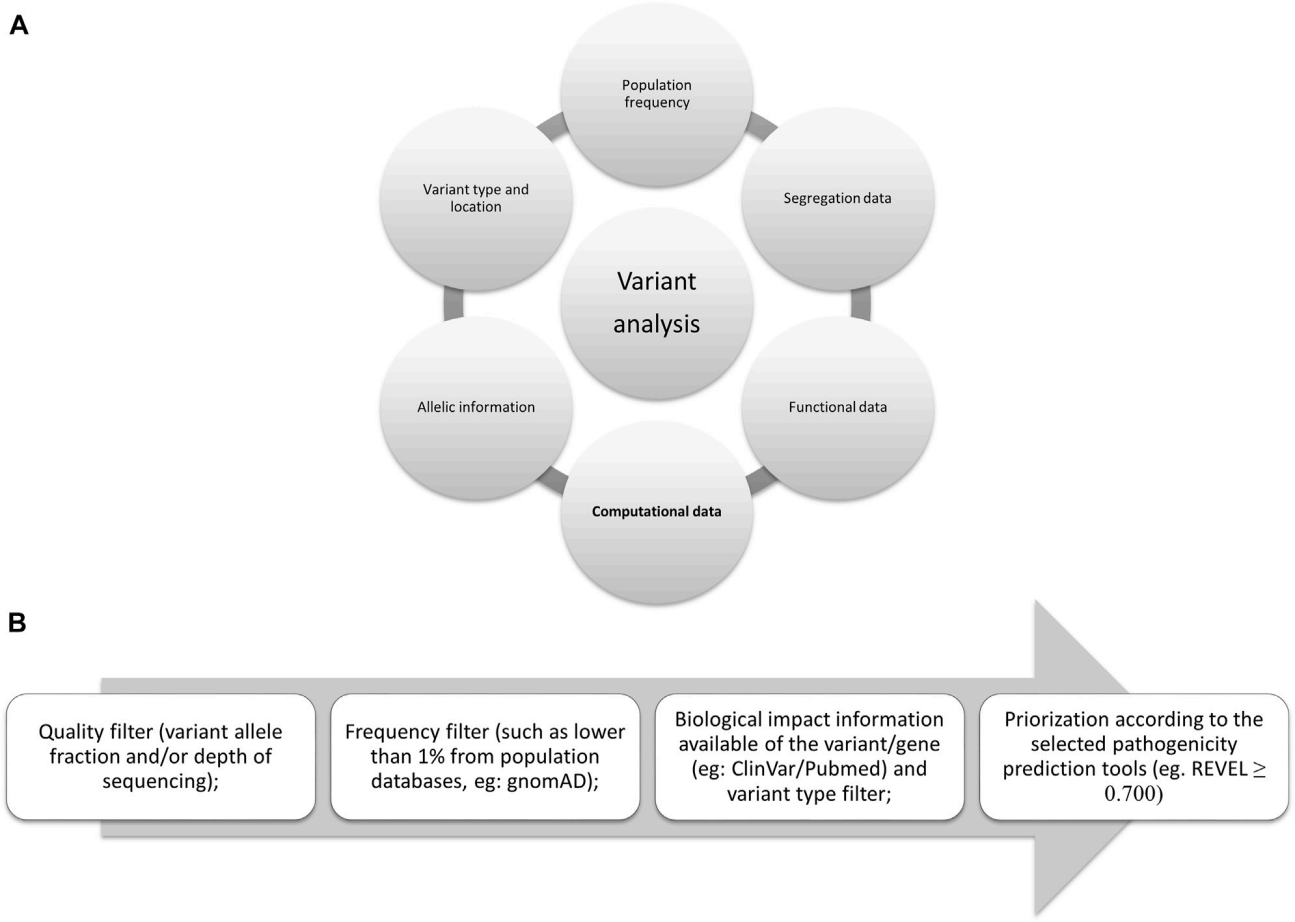
Evidences proposed by the international consortiums: **(A)** Summarized evidence of the criteria proposed by them. **(B)** Flowchart for filtering variants; *in silico* tools scores may vary, here we present the one proposed by the authors (Ioannidis et al., 2016), although ClinGen suggests higher than 0.800 for oncogenicity (Horak et al., 2022).

In 2015, several parameters were proposed by the American College of Medical Genetics and Genomics (ACMG) (Richards et al., 2015) to be used to evaluate the pathogenicity of germline variants and one of the most widely applicable parameters is *in silico* analysis. This same analysis is also included in the guidelines for somatic variants as recommended in 2017 by the Association for Molecular Pathology, the American Society of Clinical Oncology, and the College of American Pathologists (Li et al., 2017) and more recently, in 2022, by the Clinical Genome Resource (ClinGen), Cancer Genomics Consortium (CGC), and Variant Interpretation for Cancer Consortium (VICC) (Figure 1A) (Horak et al., 2022).

The delicate process of variant classification requires several levels of evidence (from supporting to very strong evidence, according to the last proposal from ClinGen). To assess the candidate variants of a large-scale sequencing study, several filters should be implemented following the guidelines and evidence from the consortiums. To carefully remove variants that may not be associated with the disease in question some filters should be applied, such as sequencing quality filters, population frequency (available in gnomAD), biological and clinical information (segregation and functional data available in different databases such as COSMIC, ClinVar, or PubMed), variant location in the protein (active sites or hotspots) and variant type (synonymous, missense, frameshift, in frame, nonsense, stop-loss, splicing site), and the selected prediction tools for the variant types studied (as not all tools analyze all variant types or locations—Figure 1B).

The term “*in silico*” is an expression derived from the biological experimental terms “*in vivo*” (in the living system) and “*in vitro*” (in the test tube) and, in general, implies the acquisition of knowledge by computer simulations and model analysis, meaning the analysis or simulation of an experiment performed in a virtual environment. As a large portion of the variants in a sequencing file (whole genome or whole exome or even panels) still have unknown clinical significance, employing these *in silico* tools may facilitate efforts to characterize the variants.

We here categorized the *in silico* pathogenicity prediction tools according to the parameter we considered as a differential, or as a signature analysis in the method by which the variants are evaluated, nevertheless, they are not necessarily exclusive to one of the following groups: 1) analyzing sequence conservation in both evolutionary and interspecific contexts, 2) evaluating structural/physicochemical parameters, 3) employing supervised machine learning, 4) employing unsupervised machine learning, and 5) utilizing modifications of splicing.

Although these tools are highly complex and sophisticated, they should not be used alone to classify a variant neither be used as diagnostic parameters by themselves. The tools are only part of the very delicate classification process, because finding a pathogenic variant may imply medical intervention for a person or even their families, sometimes for a lifetime, and even influence a couple’s decision to have a child.

**FIGURE 2:**
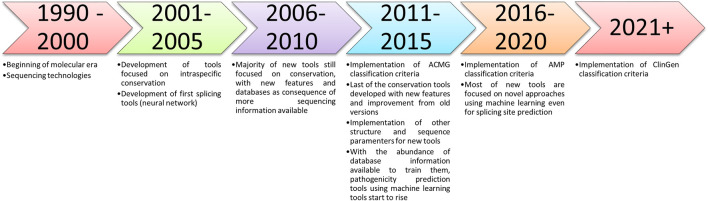
Timeline of the described *in silico* tools methods and the criteria implemented suggesting their use.

The aim of this paper is not to analyze and propose the best *in silico* pathogenicity prediction tools but to describe or catalog the well-established and recently developed tools (e.g., machine learning and ensemble methods—Figure 2), that can be used to analyze variants and help in the researcher decision.

## *In silico* pathogenicity prediction tools

### Analysis of interspecific and evolutionary sequential conservation

Conserved regions of DNA usually contain information that is crucial for the homeostasis of the cellular environment and the entire body itself. If that region encodes a protein, variants located in that position may have a pathogenic impact on the structure and consequently on the role/function of that protein (Mooney and Klein, 2002).

To evaluate the pathogenicity of variants, the following tools examine the conservation of the region with a variety of mathematical and biochemical methodologies to score how much that variant altered a conserved region; some of these tools require data entry or use one to several sequences (from different species) to compare the conservation of the region and to predict the variant effect score, thereby increasing the probability of human error or bias, because the sensitivity of these tools relies on the amount and type/species of sequences to compare (Ng and Henikoff, 2003; Siepel et al., 2005; Stone and Sidow, 2005; Tavtigian et al., 2006; Davydov et al., 2010; Pollard et al., 2010; Reva et al., 2011; Choi et al., 2012; Shihab et al., 2013; Mi et al., 2019).

Tools in which sequence conservation is the main type of analysis are outlined below, as well as the suggested score in Table 1.

**TABLE 1:** Information about *in silico* tools for variant analysis categorized by the differential analytic features.

Software	Pathogenicity score	Link	Reference	Citations^a^
Interspecific and evolutionary sequential conservation				
SIFT	<0.05	N/A	Ng et al. (2003)	5,656
Align-GVGD	> C35	http://agvgd.hci.utah.edu/agvgd_input.php	Tavtigian et al. (2006)	678
MAPP	Not tolerated	http://mendel.stanford.edu/sidowlab/downloads/MAPP/	Stone et al. (2005)	404
PhastCons	N/A	http://compgen.bscb.cornell.edu/phast	Siepel et al. (2005)	4,252
PhyloP	N/A	http://compgen.bscb.cornell.edu/phast	Pollard et al. (2010)	2,244
GERP	≥2	http://mendel.stanford.edu/SidowLab/downloads/gerp/	Davydov et al. (2010)	1,524
Mutation Assessor	0.8 < *x* ≤ 1.9 > low impact	http://mutationassessor.org/r3/	Reva et al. (2011)	1,817
	1.9 < *x* ≤ 3.5 > medium impact			
	<3.5 > high impact			
FATHMM	<0	http://fathmm.biocompute.org.uk/	Shihab et al. (2012)	1,044
PROVEAN	−2,282	NA	Choi et al. (2012)	2,781
Panther	Deleterious	http://www.pantherdb.org/about.jsp	Mi et al. (2019)	2,356
Sequence/Structure tools				
MutPred	>0.5	http://mutpred.mutdb.org/index.html	Li et al. (2009)	807
SNPeffect	Reduced Stability	http://snpeffect.switchlab.org/	De Beats et al. (2012)	247
PolyPhen-2	Probably damaging (≥0.957)	http://genetics.bwh.harvard.edu/pph2/index.shtml	Adzhubei et al. (2013)	12,463
	Possibly damaging (0.453 ≤ *x* ≤ 0.956)			
Supervised Machine Learning Analysis				
VEST	>0.5	https://karchinlab.org/apps/appVest.html	Carter et al. (2013)	389
Mutation Taster	>0.5 (Disease Causing)	http://www.mutationtaster.org/	Schwarz et al. (2014)	3,054
Mutation Taster 2021	Deleterious	https://www.genecas2003cade.org/MutationTaster2021/	Steinhaus et al. (2021)	32
CADD	>20	https://cadd.gs.washington.edu/	Kircher et al. (2014)	5,163
M-CAP	>0.025	http://bejerano.stanford.edu/mcap/	Jagadeesh et al. (2016)	621
REVEL	>0.5	N/A	Ioannidis et al. (2016)	1,109
BayesDel	>0.0692655	http://fengbj-laboratory.org/BayesDel/BayesDel.html	Feng et al. (2017)	75
Unsupervised Machine Learning Analysis				
GenoCanyon	>0.5	http://zhaocenter.org/GenoCanyon_Index.html	Lu et al. (2015)	151
Eigen	>0.5	http://www.columbia.edu/∼ii2135/download.html	Ionita-Laza et al. (2016)	483
Splicing analysis				
Nnsplice	0.4	https://www.fruitfly.org/seq_tools/splice.html	Reese et al. (1997)	1904
MaxEntScan	N/A	http://hollywood.mit.edu/burgelab/maxent/Xmaxentscan_scoreseq.html	Yeo et al. (2004)	1959
HSF	N/A	https://www.genomnis.com/access-hsf	Desmet et al. (2009)	2,556
dbscSNV	0.6	http://www.liulab.science/dbscsnv.html	Jian et al. (2014)	365
SpliceAI	0.5	https://spliceailookup.broadinstitute.org/	Jaganathan et al. (2019)	826

^a^ Number of citations according to Google Scholar.

### Sorts intolerant from tolerant (SIFT)

The SIFT tool was designed by John Craig Venter Institute in 2003, and at present, it is only available through annotation software [such as Ensembl Variant Effect Predictor (VEP) (McLaren et al., 2016) or ANNOVAR (Wang et al., 2010)]. This tool compares the submitted sample with several similar sequences. SIFT analyzes all the possible amino acid substitutions for the inputted sequence and classifies them as tolerated, whereas the amino acid change is predicted to not compromise the protein’s function, or not tolerated, the pathogenic prediction implying a possible altered function. The classification is performed using Bayes (probabilistic theorem) supplemented by Dirichlet [distributive for analysis of unknown results from Bayesian analyses (Ng and Henikoff, 2003)].

### Mutation assessor

This tool was designed by Memorial Sloan Kettering Cancer Center (cBio@MSKCC) in 2011 and is available for annotation software and on the website http://mutationassessor.org/r3/. The tool extracts alignment information from protein families of large numbers of homologous sequences grouped into aligned sets (families and subfamilies) and explores 3D structures of homologous sequences. These structures are evolutionarily (through conservation and entropy) compared with the mutated protein. This approach generates a low, medium, and high risk regarding the deleterious impact of the substituted amino acid protein function (Reva et al., 2011).

### Align-GVGD

The tool was designed by Tavtigian et al. (2006) in the International Agency for Research on Cancer (IARC) in 2006 but is now housed and available only through Huntsman Cancer Institute (University of Utah)’s website (http://agvgd.hci.utah.edu/agvgd_input.php). This tool compares the protein evolutionarily by two indices, the Grantham variation and deviation (GV and GD, respectively). The minimum and maximum values of composition, polarity, and volume are used as coordinates of a plane on different axes forming a “box.” The GV index is calculated by Euclidean distance (distance following the Pythagorean theorem parameters) from the main diagonal of the normal protein boxes (evolutionary chain), while the GD index is calculated by plotting the mutation and determining the Euclidean distance from the normal protein box to the mutated one. In other words, the larger the GD is, and the smaller the GV is, the greater the likelihood that the amino acid substitution will be pathogenic. The result is a table classification ranging from C0 (low chance of being deleterious) to C65 (probably deleterious) with the association of the two quantities.

### Multivariate analysis of protein polymorphism (MAPP)

The tool was designed by Stanford University in 2005 and can only be used through command lines. This tool compares hydrophobia, polarity, charge, volume, free energy in an alpha helix conformation, and free energy in a beta chain conformation among species submitted and predicts the impact that a variant causes by analyzing the conservation of these parameters in the protein. By testing each possible amino acid substitution, the results are classified into tolerable and intolerable substitutions (Stone and Sidow, 2005).

### Protein analysis through evolutionary relationships (PROVEAN)

The tool was designed in the Thomas laboratory at the University of Southern California in 2003 and later updated in 2013, being available only through its website (http://www.pantherdb.org/about.jsp). This tool has several implementations that combine factors such as complete organisms genomes, gene function classification, pathway analysis, and statistical tools (hidden Markov models) to analyze several parameters of sequencing and genetic information. Pathogenicity variant prediction functions by estimating the likelihood that an encoding nonsynonymous single nucleotide polymorphism will have a functional impact on the protein by calculating the length of time (in millions of years) of a given amino acid being preserved in the protein of interest. The longer the preservation time of the region or the reference allele, the greater the chance of functional impact (Mi et al., 2019).

### Functional analysis through hidden Markov models (FATHMM)

This tool was designed by the University of Bristol in 2012 and is available for annotation software and on the website (http://fathmm.biocompute.org.uk/). The tool combines a species-independent method where homologous sequences are automatically collected [from UniRef90 (Suzek et al., 2007)], aligned, built in a hidden Markov model, and matched (lowering the human intervention). Also, sequences from the manually curated databases SUPERFAMILY (Wilson et al., 2009) and PFAM (Punta et al., 2012) are analyzed to capture important sites (important structures, domains, and conserved regions) with species-specific weightings derived from relative frequencies of disease-associated and functionally neutral amino acids mapping onto conserved protein domains to predict the functional impact of protein variants. The lower the score (<0) is, the more deleterious the variant is (Shihab et al., 2013).

### Genomic evolutionary rate profiling (GERP)

GERP was developed by the Sidow Lab with Stanford University and is available for annotation software and download on its website (http://mendel.stanford.edu/SidowLab/downloads/gerp/). GERP relies on multiple alignments by calculating the position-specific constraint score and also the significant aggregation of the segments using a continuous-time Markov process and maximum likelihood. GERP’s score ranges from −12.3 to 6.17, the higher the score, the more conserved that nucleotide/region and more likely to be deleterious (Davydov et al., 2010).

### Protein variation effect analyzer (PROVEAN)

PROVEAN was developed by the John Craig Venter research institute in 2012 and is available for annotation software. PROVEAN is one of the few tools not limited to SNV, but can also analyze in-frame variants. PROVEAN analysis consists basically of two steps: collecting and clustering sequences from NCBI NR (protein database) through BLASTP. The algorithm CD-HIT, then, removes redundant sequences and up to the 45 first most similar to the entry sequence are clustered. Secondly, a delta score is calculated for each of the clustered sequences by the BLOSUM62 substitution matrix algorithm. Finally, an average delta score of each cluster is calculated and the PROVEAN score is generated. PROVEAN score ranges from −12 to 4 with a threshold of −2,282: the lowest the score the more likely deleterious the variant although it generates a prediction between deleterious and neutral (Choi et al., 2012).

### Phylogenetic analysis with space/time/conservation models (PhastCons)

PhastCons was developed by the University of California, Pennsylvania State University, Washington University School of Medicine, and Baylor College of Medicine in 2005. It is available through annotations software and as part of the PHAST package (http://compgen.bscb.cornell.edu/phast). PhastCons works on a phylogenetic hidden Markov model and maximum likelihood (using an expectation-maximization algorithm). It uses multiple alignments from several species considering the individual and the flanking columns of the alignments. It ranges from 0 to 1 and the higher the score, the more conserved the region (Siepel et al., 2005).

### Phylogenetic P-values (PhyloP)

PhyloP was developed by the University of California and Cornell University in 2010 and is available through annotation software and also available as part of the PHAST package (http://compgen.bscb.cornell.edu/phast). To generate its score, PhyloP considers four tests in its phylogenic model (of 46 genomes): a likelihood ratio test, a score test (Fisher information matrix and Monte Carlo), an exact distribution of numbers of substitutions based test, and the genomic evolutionary rate profiling (GERP) test. PhyloP’s score ranges from −20 to 9.631 and the higher the score the more conserved the region (Pollard et al., 2010).

## Sequence or structural protein alteration

Sequence conservation itself is important for a protein to keep functioning, but is not the only criterion. The structure must be stable enough for the protein to perform its activity. Some variants may not be located in conserved regions; therefore, they may not be detected as deleterious by some of the tools cited previously that only/mainly rely on conservation, but they could still disrupt the cores, active sites, or important components of the protein (Li et al., 2009; De Baets et al., 2012; Adzhubei et al., 2013). The following tools have, among other features, structural parameters as differential criteria in their analyses for variant classification, and, for this reason, some of them require specific knowledge to interpret the results properly. The proposed scores are located in Table 1.

### Polymorphism phenotyping-2 (PolyPhen-2)

This tool was developed by Harvard in 2010 and is available for annotation software and online (http://genetics.bwh.harvard.edu/pph2/index.shtml). The tool assesses sequence features on how fundamental the location where the variant is (such as active or binding sites). It also uses the 3D protein mapping on Protein Structure Database (PDB) to assess the conservation of the input sequence. And how much the variant changed parameters, such as accessible area, hydrophobia, chemical-electrostatic interactions, secondary structure conformation, solvent-accessible surface area, and Phi-Psi dihedral angles. The Naïve Bayes classifier of PolyPhen-2 was trained with supervised machine learning using pathogenic and non-damaging alterations from UniProtKB(28).

Despite having conservation and supervised machine learning in its methodology, the structure features used by PolyPhen-2 were the differential to present in this section. After calculating these parameters by submitting the protein with the mutated amino acid, the probability that this mutation is deleterious is estimated by Bayes, and the score is converted into classes: probably benign, possibly benign, possibly damaging, or probably damaging (Adzhubei et al., 2013).

### SNPeffect

SNPeffect was developed by the VIB Switch Laboratory in 2011 and is only free for academic purposes (http://snpeffect.switchlab.org/). This tool has a database containing four algorithms: TANGO, which detects regions prone to aggregation in protein sequences by analyzing hydrophobia and propensity of beta-leaf formation; WALTZ, which accurately and specifically predicts the regions of amyloid formation in protein sequences; LIMBO, which is a chaperone binding site predictor for Hsp70 chaperones; and FoldX, which calculates the mutation free energy difference through structural information. Functional sites and structural characteristics, cell processing, posttranslational modification, and domain annotation are derived from studies performed by other researchers and databases such as PFAM (Punta et al., 2012). Each algorithm produces a score, and their association indicates how altered the protein function was (De Baets et al., 2012).

### MutPred

The tool was developed and is maintained by Indiana University, the University of Washington, and the University of California San Diego in 2009 (available at http://mutpred.mutdb.org/index.html). The methods for this tool were inspired by SIFT but it was improved for human diseases based upon protein sequence, changes in structural features, and functional sites between wild-type and mutant sequences. This tool was upgraded with the addition of gain/loss of 14 different structural and functional properties and an evolutionarily conservative calculation. The tool was trained using HGMD deleterious mutations and neutral polymorphisms from the Swiss-Prot protein database. Despite being trained, the tool has several structural parameters differentiating itself, maintaining it in this section. The training dataset has been updated to contain 39,218 HGMD (Stenson et al., 2009) disease-associated mutations and 26,439 putatively neutral Swiss-Prot (Boeckmann et al., 2003) substitutions. A new version was updated in 2020 using neural network, also trained with 53,180 pathogenic variants and 2,06,946 (putatively) neutral variants from HGMD, SwissVar and dbSNP. This tool provides an empirical *p*-value of a possible altered biological parameter; if it is significant, that parameter is altered (Li et al., 2009; Pejaver et al., 2020).

## Supervised machine learning analysis

Supervised machine learning is a system based on neural networks, random forests, support vector machines, and mathematical/statistical classifiers. These tools need to be “trained” with variants already associated and not associated with disease for the software to “learn” how to predict pathogenicity. This category utilizes several computational, mathematical, and biochemical parameters that could not be captured by tools focused on conservation or structure only, but they require databases to be trained; therefore, the databases must be curated, and the larger the amount of data available is, the better the tool will be trained, and consequently, more accurate the classification will be. All suggested scores are presented in Table 1.

### MutationTaster

MutationTaster was one of the first tools to analyze intronic, synonymous, and short indels. This tool was developed by Universitätsmedizin Berlin and Cardiff University in 2014 and is available for annotation software and on its website (mutationtaster.org). To predict the variant effect, the tool contains single nucleotide polymorphisms and deletions from the 1000 Genomes Project (Genomes Project et al., 2012) and pathogenic variants found in ClinVar and the HGMD (Stenson et al., 2014). Variants common (more than four times in homozygous genes) on 1000 Genomes/HapMap are automatically neutral, while pathogenic variants in ClinVar are automatically disease-causing. In addition, regulatory test data from the ENCODE (Consortium, 2012) and JASPAR7 (Portales-Casamar et al., 2010) projects have been integrated along with evolutionary conservation scores on DNA variants. This tool also uses a Grantham standard splicing analyzer tool that provides comparative biochemical measurements of amino acids according to their polarity, volume, and composition in addition to the internal implementation of the NNsplice tool (Reese et al., 1997). Through all this database information, the tool estimates Bayes on disease-causing or polymorphisms (Schwarz et al., 2014).

A new version of MutationTaster has been released in 2021 and is currently available on its website (https://www.genecascade.org/MutationTaster2021). Due to being recent, it has not been tested against other tools yet. Other than a friendlier interface, several major changes were implemented, such as adapting the tool for next-generation sequencing data, updating new variants (including rare variants) from gnomAD, ClinVar, and HGMD Professional with conservation information on the tool training set, and implementing gnomAD to remove homozygous benign variants that occur in healthy individuals. Besides, they included new databases for variant pathogenicity information [Ensembl (Howe et al., 2021) and UniProt], splicing prediction was changed from NNsplice to MaxEntScan, pLI scores (tolerance of a gene to loss of function considering the amount of truncating variants) (Ziegler et al., 2019) from ExAC and integration of MutationDistiller (disease phenotype analysis) were included. Mutation Taster now does not use the Naïve Bayes classifier anymore, a new model using Random Forest has been implemented to improve the results which are now binary: deleterious or benign (Steinhaus et al., 2021).

### Combined annotation dependent depletion (CADD)

The tool was developed by the Berlin Institute of Health, Universitätsmedizin Berlin, the University of Washington, the HudsonAlpha Institute for Biotechnology, and the Brotman Baty Institute for Precision Medicine in 2018. The tool is available for annotation software and online (https://cadd.gs.washington.edu/). This tool was designed to score most single nucleotide variants or small insertions/deletions using a support vector machine; it measures the deleteriousness of the variant according to molecular functionality and pathogenicity. To classify the variants, annotation is performed with the software VEP (SIFT and PolyPhen-2), with conservation scores from PhasCons, phyloP, and Gerp++, plus some data from gene expression values, acetylation, methylation, nucleosome occupancy, chromatin status, genomic studies, transcription factors, 1,000 Genomes, and Exome Sequencing Project (ESP) (Fu et al., 2013) frequency and Granthan scores (Grantham, 1974). CADD’s support vector machine was trained using several public databases (such as ClinVar and Exome Sequencing Project) and data from the literature’s study databases. A higher score indicates a higher chance of deleteriousness, and the authors suggest a cutoff between 10 and 20 (Kircher et al., 2014).

### Variant effect scoring tool (VEST)

The tool was developed by Johns Hopkins University in 2012 and is available through annotation software and can be downloaded from its server (https://karchinlab.org/apps/appVest.html). VEST uses the supervised machine learning Random Forest systematic (its “forest” containing 1,000 “trees”): all features were standardized with the Z-score, and the tool was trained with variants from HGMD (Professional v2012.2) and ESP; these mutations were annotated with 86 available functional features. For functional prediction, this tool uses statistical hypothesis testing from CHASM, which is also a random forest classifier (its “forest” containing 500 “trees”) trained with variants from several sequencing studies available in the literature; the system was implemented with new parameters from the original random forest methodology and with 49 prediction features. A last gene score prediction was also implemented to predict whether the malfunctioning of that gene and its mutation results in disease and the score was generated with Fisher’s Method and Stouffer’s Z-score. The VEST final score ranges from 0 to 1, and the higher the score is, the more deleterious the variant is (Carter et al., 2013).

### Mendelian clinically applicable pathogenicity (M-CAP)

The tool was developed by Stanford University in 2016 and is available for annotation software and on the website (http://bejerano.stanford.edu/mcap/). Developed with the ensemble method, this tool employs pathogenicity scores from nine prediction tools [CADD, SIFT, PolyPhen-2, MutationTaster, MutationAssessor, FATHMM, LRT, MetaLR, and MetaSVM (Dong et al., 2015)] and incorporates seven established measurements of base pairs, amino acids, genomic regions, and gene conservation (GERP ++, RVIS, PhyloP, PhastCons, PAM250, BLOSUM62, and SIPHY), exhibiting 95% sensitivity. The tool also incorporates 298 new parameters derived from multiple sequence alignments of 99 primates, mammals, and vertebrates with the human genome. The higher the score is (>0.025), the more deleterious the variant is (Jagadeesh et al., 2016).

### Rare exome variant ensemble learner (REVEL)

The tool was developed by a collaboration among more than 25 research centers and is available through annotation software and by its precomputed scores (https://sites.google.com/site/revelgenomics/). This tool is designed in the ensemble method that integrates 18 pathogenetic prediction scores from 13 tools (GERP ++, SIFT, PolyPhen-2, MutationTaster, Mutation Assessor, FATHMM, LRT, PhyloP, PhastCons, SIPHY, MutPred, PROVEAN, and VEST) with eight sequence conservation scores and ten functional scores. REVEL was also trained with rare pathogenic variants [SwissVar (Mottaz et al., 2010) and ClinVar], excluding those already used in one of the included tools. The score varies according to the preference of the user: a more sensitive deleterious score is higher than 0.5, and a more specific deleterious score is higher than 0.7 (Ioannidis et al., 2016); a recent study suggests using a score higher than 0.8 for deleteriousness and lower than 0.4 for benign variants (Wilcox et al., 2021).

### BayesDel

This tool was developed by the University of Utah in 2018 and is available through its software (http://fengbj-laboratory.org/BayesDel/BayesDel.html). The tool is designed with the ensemble method integrating the pathogenicity scores from several tools, including PolyPhen-2, SIFT, FATHMM, LRT, MutationTaster, Mutation Assessor, PyloP, GERP++, and SiPhy. To combine the pathogenicity predictors for each of them, likelihood ratios were created, and a naïve Bayesian model was subsequently applied. To train the tool, pathogenic variants were obtained from ClinVar and UniProtKB (excluding variants from the ENIGMA dataset), and neutral variants were obtained from the UniProtKB, dbSNP, 1000 Genomes Project, Exome Aggregation Consortium (EXAC), ALSPAC, and TWINSUK cohorts (UK10K Project). The score ranges from −1.29334 to 0.75731, and the higher the score is, the more deleterious the variant (Feng, 2017).

## Unsupervised machine learning analysis

The unsupervised tools may also be based on the same systems as the supervised ones, although they do not rely on extensive databases to be trained, thereby avoiding any bias that may be associated with them. Theoretically, supervised tools are more reliable than unsupervised ones when researchers have a proper training database, but if the information being researched is new or there is little/no evidence available, unsupervised tools are preferable (Lu et al., 2015; Ionita-Laza et al., 2016). The suggested scores for unsupervised tools are presented in Table 1.

### Eigen

The Eigen tool was developed by Columbia University and the Icahn School of Medicine at Mount Sinai in 2016 and is available through its precomputed scores (http://www.columbia.edu/∼ii2135/download.html) or annotation software. This tool uses the support vector machine and is based on several annotations for a set of variants obtained from conservation tools and regulatory/functional information from studies and databases. With this information, Eigen uses the population frequency from the 1000 Genomes Project and data from the literature to calculate a meta-score classifying the variant: the higher the score, the higher the chance of the variant being pathogenic (Ionita-Laza et al., 2016).

### GenoCanyon

GenoCanyon is a whole genome unsupervised annotation tool employing statistical models with 22 computational and experimental parameters. The statistical model calculates whether the location is functional. The annotations concerning conservation or biochemical activity were downloaded from the UCSC Genome Browser and included information on genomic conservation measures (GERP and PhyloP), chromatin status, histone modifications, and transcription factor binding sites. Finally, with a total of 49 parameters, a score (higher scores have a higher chance of pathogenicity) is calculated to estimate the extent of the alteration in functionality (Lu et al., 2015).

## Splicing site alteration analysis

Most of the bioinformatics tools cited above analyze variants located on coding parts of the DNA (the exons) or the amino acid substitution; therefore, any variant outside that range may not even be classified.

Splicing is an important biological phenomenon that allows the removal of introns and attachment of exons; thus, if an error occurs, the protein may lose part of its sequence or gain new fragments, possibly resulting in loss of function.

Variants may occur in any region of the genome, although some regions are more prone to accumulate or receive damage. Splicing regions may also harbor variants able to cause exon skipping. To perform more powerful bioinformatics analysis of sequencing, splicing should not be overlooked.

The tools to analyze splicing are primarily based on sequence patterns where the spliceosome binds. The absence of these sequences in the splicing region or the presence (i.e., the creation of a new site due to an alteration) of these sequences inside an exon may affect protein function. These tools use several mechanisms to classify canonical and noncanonical alterations. Most of them rely on neural network systems; thus, in this section, we present tools based on that system, based on entropy, and those that integrate both systems (Reese et al., 1997; Yeo and Burge, 2004; Desmet et al., 2009; Jian et al., 2014; Jaganathan et al., 2019). Data for these tools are also presented in Table 1.

### NNSplice

This tool, which was developed by Lawrence Berkeley National Laboratory and the University of California in 1997, is one of the oldest and was used as the basis for other tools (available in: https://www.fruitfly.org/seq_tools/splice.html). NNSplice uses a generalized hidden Markov model in which it states corresponds to a gene feature (such as donor/acceptor site, intron/exon, start/end, and UTR). To capture these features (states), the tool uses identifying sensors (sensors containing data from multiple sources) where those features/states occur and infer a likelihood of alteration. Splice site recognition occurs through a neural network trained to recognize donor and acceptor sites. The detection score ranges from 0 to 1: lower scores indicate that the region is lost or is not a splicing site, while higher scores indicate a probable splicing site or the formation of a new splicing site (Reese et al., 1997).

### SpliceAI

The construction of the SpliceAI tool involved the Illumina Artificial Intelligence Laboratory, the University of California, Stanford, Massachusetts Institute of Technology, and Harvard. It was developed in 2018 and is available online (https://spliceailookup.broadinstitute.org/). SpliceAI relies on a deep neural network with 32 sequencing recognizing layers as an *in silico* model of the spliceosome to predict splicing site and crypt splicing variants. It was trained with the database GENCODE-annotated (Harrow et al., 2012) pre-mRNA transcript sequences. A delta score is generated after several equations comparing the variant and several sequences and its surroundings, it ranges from 0 to 1, and the higher the score the more likely the variant affects the splicing (local or surroundings); the authors recommend a threshold score of 0.5 (Jaganathan et al., 2019).

### Database for splicing consensus regions single nucleotide variants (dbscSNV)

The dbscSNV was developed by the University of Texas in 2014 and is available through its software or annotated database for download (http://www.liulab.science/dbscsnv.html) and on annotation software. It consists of an ensemble splicing method: the authors tested the accuracy, sensitivity, specificity, positive predictive value (PPV), and negative predictive value (NPV) of several splicing tools/models (Position Weight Matrix (PSM), MaxEntScan, NNSplice, GeneSplicer, Human Splicing Finder (HSF), NetGene2, GENSCAN, and SplicePredictor) with 13,000 variants; after checking their performance, those with better performance (PSM, MaxEntScan, HSF, and NNsplice) were selected. To improve the performance, conservation scores (phyloP) and whole-genome functional scores (CADD) were implemented. Thus, both ensemble methods (Adaboost and random forest) generate one pathogenicity prediction score each. The authors suggest a cutoff of at least 0.6 for splicing altering variants for both methods (Jian et al., 2014).

### MaxEntScan

This tool was developed by the Massachusetts Institute of Technology in 2004 (available in: http://hollywood.mit.edu/burgelab/maxent/Xmaxentscan_scoreseq.html). The tool is based on the maximum entropy (measure of disorder or randomness) principle (MEP) for short sequence motifs: calculated by Shannon entropy, which is a measure of the average uncertainty in a random variable; or by the principle of minimum relative entropy (MRE), as minimizing MRE is equivalent to maximizing the MEP. To generate a final score, several entropic formulas are applied to the input data: the higher the score is, the more likely that the region has or is a true/strong splice site (Yeo and Burge, 2004).

### Human splicing finder

The Human Splicing Finder tool (https://www.genomnis.com/access-hsf) was developed by INSERM in 2008. It combines several different algorithms to identify and predict the effects on splicing sites, including donors and receptors, ramification points, and auxiliary sequences that raise or diminish splicing: exonic splicing enhancers or silencers. These algorithms are based on position weight matrix, entropy, and motif (nucleotide sequences with biological meaning) comparisons. For each of these algorithms, consensus values and a score variation limit are defined based on literature data. The tool generates interpretations for the alteration analyzed for each relevant algorithm showing why the submitted variant is altering the splicing and one final say as altering or not altering splicing (Desmet et al., 2009).

## Which tool to use?

After understanding the mechanisms of the tools, they must be used according to the purpose of the research; therefore, there is not an optimal tool for everything. As the tools are computational systems, algorithms, and software designed to execute codes contacting mathematical and statistical models regarding biological and biochemical parameters to calculate the probability of an alteration to nullify the gene product. The result may not truly coincide with the true conditions, but the result is not “wrong”; rather, it is simply what that mathematical model indicates. Therefore, choosing the right tools and understanding their functionality is important because, as noted above, the tools employ different types of analysis, and understanding these analyses is essential to understand why a variant is or is not deleterious (some pros and cons observed are available on Supplementary Table S1). As this paper is focused on variants related to diseases, such as cancer, we selected some studies that tested these tools for that purpose or a similar one.

Grimm et al. (2015) evaluated 10 tools in five datasets: PolyPhen-2, SIFT, FATHMM (weighed and unweighed), MutationTaster, Mutation Assessor, CADD, LRT, phyloP, and GERP++. To that end, these researchers extracted variants from the datasets HumVar, ExoVar, VariBench, predictSNP, and SwissVar and separately analyzed Condel and Logit after performing measurements of true/false negatives/positives by accuracy, precision, recall/sensitivity, specificity, F-score, NPV and Matthews correlation coefficient (MCC). The researchers concluded that FATHMM (weighed) had the best performance.

In 2015, Dong et al. (2015) evaluated the performance of 18 tools: PolyPhen-2, SIFT, MutationTaster, Mutation Assessor, FATHMM, LRT, PANTHER, PhD-SNP, SNAP, SNPs&GO, MutPred, GERP++, SiPhy, PhyloP, CADD, PON-P, KGGSeq, and CONDEL. These researchers used one training set and three databases for their analysis: UniProt for training, information from Nature Genetics publications, VariBench, and the Cohorts for Heart and Aging Research in Genomic Epidemiology (CHARGE) databases for testing. After analyzing associations with false/true positives/negatives (by sensitivity and specificity), the researchers concluded that FATHMM had the best discriminative power followed by KGGSeq, an ensemble tool.

Ghosh, Ninad, and Plon evaluated the performance of 25 algorithms in 2017: CADD, Condel, DANN, EA, Eigen, FATHMM, GenoCanyon, GERP++, hEAt, integrated_fitCons, LRT, M-CAP, MetaLR, MetaSVM, Mutation Assessor, MutationTaster, Mutpred, phastCons100way, phyloP100way, Polyphen2, PROVEAN, REVEL, SIFT, and SiPhy. For the evaluation, these researchers obtained 14,819 missense variants (7,346 benign and 7,473 pathogenic) from ClinVar. Due to the blank values, as not all tools predict all variants, and to minimize bias, the authors created a subset containing 8,386 variants, which were the ones obtained from the 14,819 variants that all tools could predict. Additionally, these researchers converted all the tools’ scores into two categories only: pathogenic and benign. It was observed that 773 of the 7,346 benign variants (10.5%) were wrongly classified by all tools as pathogenic versus 64 of the 7,473 pathogenic variants (0.8%). For the performance analysis (based on sensitivity and specificity), several datasets were analyzed by the tools and estimated by the AUC (>0.90) of a receiver operator characteristic (ROC) curve. REVEL outperformed in all tests and slightly behind VEST3 followed by MetaSVM and MetaLR, which failed in only one of the 16 tests. The authors recommend using meta predictors (new ensemble and machine learning tools) alone if necessary, as they achieved the highest performance, and not simply selecting tools when they all (even meta predictors) agree on whether variants are benign or pathogenic, as suggested by the ACMG guidelines (Ghosh et al., 2017).

Li et al. (2018) performed measures of 23 tools in 2018: FATHMM, fitCons, LRT, Mutation Assessor, MutationTaster, PolyPhen-2 (HDIV), PolyPhen-2 (HVAR), PROVEAN, SIFT, VEST3, GERP++, phastCons, phyloP, SiPhy, CADD, DANN, Eigen, FATHMM-MKL, GenoCanyon, M-CAP, MetaLR, MetaSM, and REVEL. These researchers utilized three datasets to compare pathogenic and benign variants from ClinVar and pathogenic/likely pathogenic and benign/like benign variants from the IARC TP53 database (Kato et al., 2003; Bouaoun et al., 2016) and International Cancer Genome Consortium (http://icgc.org/), and the last dataset was obtained from a large-scale study (Majithia et al., 2016). After measuring the performance rate of the tools’ true and false positives and negatives (by sensitivity, specificity, positive predictive value (PPV), NPV, false positive rate (FPR), false negative rate (FNR), accuracy and MCC), the researchers concluded that REVEL and VEST3 outperformed the rest of the tools discriminating the variants on most tests and PROVEAN had a better performance on somatic and experimentally validated variants than with germline ones. However, the authors also suggest that the other tools should not be simply discarded because some of them have the advantage of predicting certain regions, such as noncoding or regulatory variants, that others do not.

In 2019, Hassan et al. (2019) analyzed the performance of eight tools, that is, FATHMM, SIFT, PROVEAN, iFish, Mutation Assessor, PANTHER, SNAP2, and PON-P2, using a dataset composed of 2,144 pathogenic variants and 3,777 neutral variants extracted from the Varibench database (http://structure.bmc.lu.se/VariBench/GrimmDatasets.php). After testing the true and false positive and negative rates (sensitivity, specificity, positive and negative likelihood ratio, PPV, NPV, and accuracy) of the tools, these researchers concluded that FATHMM outperformed the other tools evaluated.

In 2019, Niroula and Vihinen (2019) analyzed how well 10 selected *in silico* tools performed in detecting benign variants using common variants extracted from 10 subpopulations from the EXAC database (from the interval 1% ≤ frequency < 25%): CADD, FATHMM, LRT, Mutation Assessor, MutationTaster, PolyPhen-2, PROVEAN, SIFT, VEST, and PON-P2. Although PON-P2 had the highest number of unclassified variants, it also had the best specificity followed by VEST and FATHMM, which exhibited similar performance, and PROVEN, while the others exhibited similarly poorer performances in recognizing benign variants.

Tian et al. (2019) used 4,094 variants in 66 clinically relevant genes extracted from the ClinVar database in 2019 to compare SIFT and PolyPhen-2 and to evaluate five meta predictors: REVEL, BayesDel, CADD, Meta-SVM, and Eigen. Using sensitivity analysis from true/false and negative/positive data associations (PPV, NPV, and yield rate for calculating overall prediction performance), REVEL and BayesDel outperformed the other three meta predictors and surpassed SIFT and PolyPhen-2 agreements in all tests performed by the study.

More recently, in 2021, Wilcox et al. (2021) also tried to understand the applications of the ACMG criteria regarding the *in silico* tools PP3 (supporting evidence for pathogenic predictions) and BP4 (supporting evidence for benign predictions). They analyzed how frequently PP3 and BP4 were used in 727 variants curated by Clinical Genome Resource expert groups. They optimized the thresholds and among the four tools used (MPC, VEST, REVEL, and FATHMM) the authors found VEST and REVEL perform the best. The authors conclude that the data provided in their article “*provide robust, quantitative evidence that in silico predictors, when properly calibrated, can provide evidence at the supporting or, in some cases, moderate level for pathogenicity classification.*”

**FIGURE 3:**
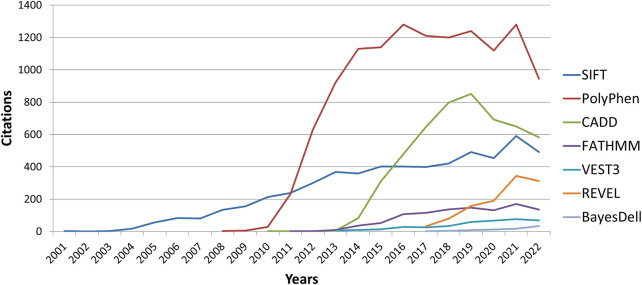
Graphic illustrating new citation number per year (according to Google Scholar) of the top three most cited tools (PolyPhen-2, SIFT, and CADD) *versus* the four tools that had frequent outperforming analysis (VEST3, REVEL, FATHMM, and BayesDel).

In this study, we described some of the most commonly employed tools for *in silico* analysis in the genomic research setting. As shown by several studies, VEST3 outperforms several tools, as do FATHMM and REVEL, although their use is slowly rising (Figure 3). Based on the characteristics of the tool as well as its performance in several studies in the literature, REVEL can be considered one of the best variant analysis tools available. It has been demonstrated to frequently outperform others and it combines VEST and FATHMM (two of the tools that frequently outperformed other tools) within its algorithm. Similarly does Bayesdel, which also outperformed several other tools. Three of them are among the newest developed tools (supervised machine learning) and compile several predictors inside their classification algorithm (ensemble method), as well as mathematical (statistics), computational, biological, and biochemical parameters.

Currently, there is no standardized method to use these tools, the consortiums advice using them with care, and advices to use as supporting evidence and that they should not be counted independently. Even so, Wilcox et al. (2021) shows that their evidence may be considered stronger if they are properly calibrated, and Ghosh et al. (2017) demonstrate that their use should be considered individually (especially for meta-predictors) if necessary, not in series, highlighting the importance of *in silico* prediction tools on variant classification and their further understanding. Perhaps, with the new tools developed by ensemble methodology, a new form of interpretation may be implemented, for instance considering these new outperforming meta-predictors (such as REVEL or BayesDel) instead of all the other tools (as they are already in use “inside” these new predictors), which may be redundant to consider a tool that is already being considered inside another one.

## Conclusion

Bioinformatics is a notably new field of research, and as with any new field, it is constantly evolving. *In silico* analysis is only one parameter for variant classification according to the three last proposals from international consortiums for variant analysis criteria.

Sequencing and variant analysis can identify several lifetime problems and disease predispositions, but it requires a specialized team, high-tech material, and computational processing power. With advances in molecular biology, these techniques are becoming less expensive and more accurate and may therefore become more accessible to the general population. Thus this review may help to amplify the knowledge of these *in silico* variants classification tools and, together with other available information that may serve as evidence for variant analysis, it may contribute in better patient care, early disease management and hence in an increase in survival rates.

## References

[B1] Adzhubei I. Jordan D. M. Sunyaev S. R. (2013). Predicting functional effect of human missense mutations using PolyPhen-2. Curr. Protoc. Hum. Genet. 7, Unit7.20. 10.1002/0471142905.hg0720s76 PMC4480630 23315928

[B2] Boeckmann B. Bairoch A. Apweiler R. Blatter M. C. Estreicher A. Gasteiger E. (2003). The SWISS-PROT protein knowledgebase and its supplement TrEMBL in 2003. Nucleic Acids Res. 31 (1), 365–370. 10.1093/nar/gkg095 12520024 PMC165542

[B3] Bouaoun L. Sonkin D. Ardin M. Hollstein M. Byrnes G. Zavadil J. (2016). TP53 variations in human cancers: New lessons from the IARC TP53 database and genomics data. Hum. Mutat. 37 (9), 865–876. 10.1002/humu.23035 27328919

[B4] Carter H. Douville C. Stenson P. D. Cooper D. N. Karchin R. (2013). Identifying Mendelian disease genes with the variant effect scoring tool. BMC Genomics 14 (3), S3. 10.1186/1471-2164-14-S3-S3 PMC3665549 23819870

[B5] Choi Y. Sims G. E. Murphy S. Miller J. R. Chan A. P. (2012). Predicting the functional effect of amino acid substitutions and indels. PLoS One 7 (10), e46688. 10.1371/journal.pone.0046688 23056405 PMC3466303

[B6] Consortium E. P. (2012). An integrated encyclopedia of DNA elements in the human genome. Nature 489 (7414), 57–74. 10.1038/nature11247 22955616 PMC3439153

[B7] Davydov E. V. Goode D. L. Sirota M. Cooper G. M. Sidow A. Batzoglou S. (2010). Identifying a high fraction of the human genome to be under selective constraint using GERP++. PLoS Comput. Biol. 6 (12), e1001025. 10.1371/journal.pcbi.1001025 21152010 PMC2996323

[B8] De Baets G. Van Durme J. Reumers J. Maurer-Stroh S. Vanhee P. Dopazo J. (2012). SNPeffect 4.0: On-line prediction of molecular and structural effects of protein-coding variants. Nucleic Acids Res. 40, D935–D939. 10.1093/nar/gkr996 22075996 PMC3245173

[B9] Desmet F. O. Hamroun D. Lalande M. Collod-Beroud G. Claustres M. Beroud C. (2009). Human splicing finder: An online bioinformatics tool to predict splicing signals. Nucleic Acids Res. 37 (9), e67. 10.1093/nar/gkp215 19339519 PMC2685110

[B10] Dong C. Wei P. Jian X. Gibbs R. Boerwinkle E. Wang K. (2015). Comparison and integration of deleteriousness prediction methods for nonsynonymous SNVs in whole exome sequencing studies. Hum. Mol. Genet. 24 (8), 2125–2137. 10.1093/hmg/ddu733 25552646 PMC4375422

[B11] Felicio P. S. Grasel R. S. Campacci N. de Paula A. E. Galvao H. C. R. Torrezan G. T. (2021). Whole-exome sequencing of non-BRCA1/BRCA2 mutation carrier cases at high-risk for hereditary breast/ovarian cancer. Hum. Mutat. 42 (3), 290–299. 10.1002/humu.24158 33326660 PMC7898723

[B12] Feng B. J. (2017). Perch: A unified framework for disease gene prioritization. Hum. Mutat. 38 (3), 243–251. 10.1002/humu.23158 27995669 PMC5299048

[B13] Fu W. O'Connor T. D. Jun G. Kang H. M. Abecasis G. Leal S. M. (2013). Analysis of 6, 515 exomes reveals the recent origin of most human protein-coding variants. Nature 493 (7431), 216–220. 10.1038/nature11690 23201682 PMC3676746

[B14] Genomes Project C. Abecasis G. R. Auton A. Brooks L. D. DePristo M. A. Durbin R. M. (2012). An integrated map of genetic variation from 1, 092 human genomes. Nature 491 (7422), 56–65. 10.1038/nature11632 23128226 PMC3498066

[B15] Ghosh R. Oak N. Plon S. E. (2017). Evaluation of *in silico* algorithms for use with ACMG/AMP clinical variant interpretation guidelines. Genome Biol. 18 (1), 225. 10.1186/s13059-017-1353-5 29179779 PMC5704597

[B16] Grantham R. (1974). Amino acid difference formula to help explain protein evolution. Science 185 (4154), 862–864. 10.1126/science.185.4154.862 4843792

[B17] Grimm D. G. Azencott C. A. Aicheler F. Gieraths U. MacArthur D. G. Samocha K. E. (2015). The evaluation of tools used to predict the impact of missense variants is hindered by two types of circularity. Hum. Mutat. 36 (5), 513–523. 10.1002/humu.22768 25684150 PMC4409520

[B18] Harrow J. Frankish A. Gonzalez J. M. Tapanari E. Diekhans M. Kokocinski F. (2012). Gencode: The reference human genome annotation for the ENCODE project. Genome Res. 22 (9), 1760–1774. 10.1101/gr.135350.111 22955987 PMC3431492

[B19] Hassan M. S. Shaalan A. A. Dessouky M. I. Abdelnaiem A. E. ElHefnawi M. (2019). Evaluation of computational techniques for predicting non-synonymous single nucleotide variants pathogenicity. Genomics 111 (4), 869–882. 10.1016/j.ygeno.2018.05.013 29842949

[B20] Horak P. Griffith M. Danos A. M. Pitel B. A. Madhavan S. Liu X. (2022). Standards for the classification of pathogenicity of somatic variants in cancer (oncogenicity): Joint recommendations of clinical genome Resource (ClinGen), cancer genomics consortium (CGC), and variant interpretation for cancer consortium (VICC). Genet. Med. 24 (5), 986–998. 10.1016/j.gim.2022.01.001 35101336 PMC9081216

[B21] Howe K. L. Achuthan P. Allen J. Allen J. Alvarez-Jarreta J. Amode M. R. (2021). Ensembl 2021. Nucleic Acids Res. 49 (D1), D884–D891. 10.1093/nar/gkaa942 33137190 PMC7778975

[B22] Ioannidis N. M. Rothstein J. H. Pejaver V. Middha S. McDonnell S. K. Baheti S. (2016). Revel: An ensemble method for predicting the pathogenicity of rare missense variants. Am. J. Hum. Genet. 99 (4), 877–885. 10.1016/j.ajhg.2016.08.016 27666373 PMC5065685

[B23] Ionita-Laza I. McCallum K. Xu B. Buxbaum J. D. (2016). A spectral approach integrating functional genomic annotations for coding and noncoding variants. Nat. Genet. 48 (2), 214–220. 10.1038/ng.3477 26727659 PMC4731313

[B24] Jagadeesh K. A. Wenger A. M. Berger M. J. Guturu H. Stenson P. D. Cooper D. N. (2016). M-CAP eliminates a majority of variants of uncertain significance in clinical exomes at high sensitivity. Nat. Genet. 48 (12), 1581–1586. 10.1038/ng.3703 27776117

[B25] Jaganathan K. Kyriazopoulou Panagiotopoulou S. McRae J. F. Darbandi S. F. Knowles D. Li Y. I. (2019). Predicting splicing from primary sequence with deep learning. Cell 176 (3), 535–548. 10.1016/j.cell.2018.12.015 30661751

[B26] Jian X. Boerwinkle E. Liu X. (2014). *In silico* prediction of splice-altering single nucleotide variants in the human genome. Nucleic Acids Res. 42 (22), 13534–13544. 10.1093/nar/gku1206 25416802 PMC4267638

[B27] Karczewski K. J. Francioli L. C. Tiao G. Cummings B. B. Alfoldi J. Wang Q. (2020). The mutational constraint spectrum quantified from variation in 141, 456 humans. Nature 581 (7809), 434–443. 10.1038/s41586-020-2308-7 32461654 PMC7334197

[B28] Kato S. Han S. Y. Liu W. Otsuka K. Shibata H. Kanamaru R. (2003). Understanding the function-structure and function-mutation relationships of p53 tumor suppressor protein by high-resolution missense mutation analysis. Proc. Natl. Acad. Sci. U. S. A. 100 (14), 8424–8429. 10.1073/pnas.1431692100 12826609 PMC166245

[B29] Kircher M. Witten D. M. Jain P. O'Roak B. J. Cooper G. M. Shendure J. (2014). A general framework for estimating the relative pathogenicity of human genetic variants. Nat. Genet. 46 (3), 310–315. 10.1038/ng.2892 24487276 PMC3992975

[B30] Landrum M. J. Lee J. M. Benson M. Brown G. R. Chao C. Chitipiralla S. (2018). ClinVar: Improving access to variant interpretations and supporting evidence. Nucleic Acids Res. 46 (D1), D1062–D1067. 10.1093/nar/gkx1153 29165669 PMC5753237

[B31] Li B. Krishnan V. G. Mort M. E. Xin F. Kamati K. K. Cooper D. N. (2009). Automated inference of molecular mechanisms of disease from amino acid substitutions. Bioinformatics 25 (21), 2744–2750. 10.1093/bioinformatics/btp528 19734154 PMC3140805

[B32] Li J. Zhao T. Zhang Y. Zhang K. Shi L. Chen Y. (2018). Performance evaluation of pathogenicity-computation methods for missense variants. Nucleic Acids Res. 46 (15), 7793–7804. 10.1093/nar/gky678 30060008 PMC6125674

[B33] Li M. M. Datto M. Duncavage E. J. Kulkarni S. Lindeman N. I. Roy S. (2017). Standards and guidelines for the interpretation and reporting of sequence variants in cancer: A joint consensus recommendation of the association for molecular Pathology, American society of clinical Oncology, and College of American Pathologists. J. Mol. Diagn. 19 (1), 4–23. 10.1016/j.jmoldx.2016.10.002 27993330 PMC5707196

[B34] Lu Q. Hu Y. Sun J. Cheng Y. Cheung K. H. Zhao H. (2015). A statistical framework to predict functional non-coding regions in the human genome through integrated analysis of annotation data. Sci. Rep. 5, 10576. 10.1038/srep10576 26015273 PMC4444969

[B35] Majithia A. R. Tsuda B. Agostini M. Gnanapradeepan K. Rice R. Peloso G. (2016). Prospective functional classification of all possible missense variants in PPARG. Nat. Genet. 48 (12), 1570–1575. 10.1038/ng.3700 27749844 PMC5131844

[B36] McLaren W. Gil L. Hunt S. E. Riat H. S. Ritchie G. R. Thormann A. (2016). The Ensembl variant effect predictor. Genome Biol. 17 (1), 122. 10.1186/s13059-016-0974-4 27268795 PMC4893825

[B37] Mi H. Muruganujan A. Huang X. Ebert D. Mills C. Guo X. (2019). Protocol Update for large-scale genome and gene function analysis with the PANTHER classification system (v.14.0). Nat. Protoc. 14 (3), 703–721. 10.1038/s41596-019-0128-8 30804569 PMC6519457

[B38] Mooney S. D. Klein T. E. (2002). The functional importance of disease-associated mutation. BMC Bioinforma. 3, 24. 10.1186/1471-2105-3-24 PMC128831 12220483

[B39] Mottaz A. David F. P. Veuthey A. L. Yip Y. L. (2010). Easy retrieval of single amino-acid polymorphisms and phenotype information using SwissVar. Bioinformatics 26 (6), 851–852. 10.1093/bioinformatics/btq028 20106818 PMC2832822

[B40] Nakagawa H. Fujita M. (2018). Whole genome sequencing analysis for cancer genomics and precision medicine. Cancer Sci. 109 (3), 513–522. 10.1111/cas.13505 29345757 PMC5834793

[B41] Ng P. C. Henikoff S. (2003). Sift: Predicting amino acid changes that affect protein function. Nucleic Acids Res. 31 (13), 3812–3814. 10.1093/nar/gkg509 12824425 PMC168916

[B42] Niroula A. Vihinen M. (2019). How good are pathogenicity predictors in detecting benign variants? PLoS Comput. Biol. 15 (2), e1006481. 10.1371/journal.pcbi.1006481 30742610 PMC6386394

[B43] Pejaver V. Urresti J. Lugo-Martinez J. Pagel K. A. Lin G. N. Nam H. J. (2020). Inferring the molecular and phenotypic impact of amino acid variants with MutPred2. Nat. Commun. 11 (1), 5918. 10.1038/s41467-020-19669-x 33219223 PMC7680112

[B44] Pollard K. S. Hubisz M. J. Rosenbloom K. R. Siepel A. (2010). Detection of nonneutral substitution rates on mammalian phylogenies. Genome Res. 20 (1), 110–121. 10.1101/gr.097857.109 19858363 PMC2798823

[B45] Portales-Casamar E. Thongjuea S. Kwon A. T. Arenillas D. Zhao X. Valen E. (2010). Jaspar 2010: The greatly expanded open-access database of transcription factor binding profiles. Nucleic Acids Res. 38, D105–D110. 10.1093/nar/gkp950 19906716 PMC2808906

[B46] Punta M. Coggill P. C. Eberhardt R. Y. Mistry J. Tate J. Boursnell C. (2012). The Pfam protein families database. Nucleic Acids Res. 40, D290–D301. 10.1093/nar/gkr1065 22127870 PMC3245129

[B47] Reese M. G. Eeckman F. H. Kulp D. Haussler D. (1997). Improved splice site detection in Genie. J. Comput. Biol. 4 (3), 311–323. 10.1089/cmb.1997.4.311 9278062

[B48] Reva B. Antipin Y. Sander C. (2011). Predicting the functional impact of protein mutations: Application to cancer genomics. Nucleic Acids Res. 39 (17), e118. 10.1093/nar/gkr407 21727090 PMC3177186

[B49] Richards S. Aziz N. Bale S. Bick D. Das S. Gastier-Foster J. (2015). Standards and guidelines for the interpretation of sequence variants: A joint consensus recommendation of the American College of medical genetics and genomics and the association for molecular Pathology. Genet. Med. 17 (5), 405–424. 10.1038/gim.2015.30 25741868 PMC4544753

[B50] Schwarz J. M. Cooper D. N. Schuelke M. Seelow D. (2014). MutationTaster2: Mutation prediction for the deep-sequencing age. Nat. Methods 11 (4), 361–362. 10.1038/nmeth.2890 24681721

[B51] Shihab H. A. Gough J. Cooper D. N. Stenson P. D. Barker G. L. Edwards K. J. (2013). Predicting the functional, molecular, and phenotypic consequences of amino acid substitutions using hidden Markov models. Hum. Mutat. 34 (1), 57–65. 10.1002/humu.22225 23033316 PMC3558800

[B52] Siepel A. Bejerano G. Pedersen J. S. Hinrichs A. S. Hou M. Rosenbloom K. (2005). Evolutionarily conserved elements in vertebrate, insect, worm, and yeast genomes. Genome Res. 15 (8), 1034–1050. 10.1101/gr.3715005 16024819 PMC1182216

[B53] Steinhaus R. Proft S. Schuelke M. Cooper D. N. Schwarz J. M. Seelow D. (2021). Nucleic Acids Res. 49 (W1), W446–W451. 10.1093/nar/gkab266 33893808 PMC8262698

[B54] Stenson P. D. Mort M. Ball E. V. Howells K. Phillips A. D. Thomas N. S. (2009). The human gene mutation database: 2008 update. Genome Med. 1 (1), 13. 10.1186/gm13 19348700 PMC2651586

[B55] Stenson P. D. Mort M. Ball E. V. Shaw K. Phillips A. Cooper D. N. (2014). The human gene mutation database: Building a comprehensive mutation repository for clinical and molecular genetics, diagnostic testing and personalized genomic medicine. Hum. Genet. 133 (1), 1–9. 10.1007/s00439-013-1358-4 24077912 PMC3898141

[B56] Stone E. A. Sidow A. (2005). Physicochemical constraint violation by missense substitutions mediates impairment of protein function and disease severity. Genome Res. 15 (7), 978–986. 10.1101/gr.3804205 15965030 PMC1172042

[B57] Suzek B. E. Huang H. McGarvey P. Mazumder R. Wu C. H. (2007). UniRef: Comprehensive and non-redundant UniProt reference clusters. Bioinformatics 23 (10), 1282–1288. 10.1093/bioinformatics/btm098 17379688

[B58] Tate J. G. Bamford S. Jubb H. C. Sondka Z. Beare D. M. Bindal N. (2019). Cosmic: The Catalogue of somatic mutations in cancer. Nucleic Acids Res. 47 (D1), D941–D947. 10.1093/nar/gky1015 30371878 PMC6323903

[B59] Tavtigian S. V. Deffenbaugh A. M. Yin L. Judkins T. Scholl T. Samollow P. B. (2006). Comprehensive statistical study of 452 BRCA1 missense substitutions with classification of eight recurrent substitutions as neutral. J. Med. Genet. 43 (4), 295–305. 10.1136/jmg.2005.033878 16014699 PMC2563222

[B60] Tian Y. Pesaran T. Chamberlin A. Fenwick R. B. Li S. Gau C. L. (2019). REVEL and BayesDel outperform other *in silico* meta-predictors for clinical variant classification. Sci. Rep. 9 (1), 12752. 10.1038/s41598-019-49224-8 31484976 PMC6726608

[B61] UniProt C. (2019). UniProt: A worldwide hub of protein knowledge. Nucleic Acids Res. 47 (D1), D506–D15. 10.1093/nar/gky1049 30395287 PMC6323992

[B62] Walsh T. Casadei S. Lee M. K. Pennil C. C. Nord A. S. Thornton A. M. (2011). Mutations in 12 genes for inherited ovarian, fallopian tube, and peritoneal carcinoma identified by massively parallel sequencing. Proc. Natl. Acad. Sci. U. S. A. 108 (44), 18032–18037. 10.1073/pnas.1115052108 22006311 PMC3207658

[B63] Wang K. Li M. Hakonarson H. (2010). Annovar: Functional annotation of genetic variants from high-throughput sequencing data. Nucleic Acids Res. 38 (16), e164. 10.1093/nar/gkq603 20601685 PMC2938201

[B64] Wilcox E. H. Sarmady M. Wulf B. Wright M. W. Rehm H. L. Biesecker L. G. (2021). Evaluating the impact of *in silico* predictors on clinical variant classification. Genet. Med. 24, 924–930. 10.1016/j.gim.2021.11.018 34955381 PMC9164215

[B65] Wilson D. Pethica R. Zhou Y. Talbot C. Vogel C. Madera M. (2009). SUPERFAMILY--sophisticated comparative genomics, data mining, visualization and phylogeny. Nucleic Acids Res. 37, D380–D386. 10.1093/nar/gkn762 19036790 PMC2686452

[B66] Yeo G. Burge C. B. (2004). Maximum entropy modeling of short sequence motifs with applications to RNA splicing signals. J. Comput. Biol. 11 (2-3), 377–394. 10.1089/1066527041410418 15285897

[B67] Ziegler A. Colin E. Goudenege D. Bonneau D. (2019). A snapshot of some pLI score pitfalls. Hum. Mutat. 40 (7), 839–841. 10.1002/humu.23763 30977936

