# Supplementary material for: Insights on variant analysis in silico tools for pathogenicity prediction
Source: Front Genet. 2022 Nov 29;13:1010327. doi: 10.3389/fgene.2022.1010327 (PMC9774026; doi:10.3389/fgene.2022.1010327)
Supplement: Supplementary file 1 [file Table1.docx]

| Supplementary table 1 – Practicality for the tools usage | | |
| --- | --- | --- |
| **Tool** | **Pros** | **Cons** |
| SIFT | Largely used and included in several meta-predictors | Old tool without recent update, analyses missense only |
| Align-GVGD | Tool developed for some genes | Species limitation, analyses missense only |
| MAPP | Compares the conservation of several physicochemical parameters | No website to analyses, for missense only, complex input data |
| PhastCons | Largely used and included in several meta-predictors | No website to analyses, for missense only |
| PhyloP | Included in several meta-predictors | No website to analyses, for missense only |
| GERP | Included in several meta-predictors | No website to analyses, for single nucleotide variant only |
| Mutation Assessor | Included in several meta-predictors, version 3 in december/2015, uses entropy | For missense only |
| FATHMM | Outperforming tool, included in several meta-predictors | NA |
| PROVEAN | From the same institute of SIFT, showed good performance when tested for somatic and experimentally validated variants (Li et al, 2018) | No website to analyse |
| Panther | Recently updated | Requires FASTA input, for missense only |
| MutPred | Display possible altered molecular mechanisms, recently updated | Requires FASTA input, different algorithms for each type of variant |
| SNPeffect | Four algorithms for specific biochemical features | Requires FASTA (or similar data) input, for missense only, complex output data |
| PolyPhen-2 | Most cited tool | For missense only |
| VEST | Outperforming tool | For missense only |
| Mutation Taster | Display several of the variant altering mechanisms | Upgraded version available |
| Mutation Taster 2021 | Upgraded version | NA |
| CADD | Largely used meta-predictor | NA |
| M-CAP | Meta-predictor | For missense only |
| REVEL | Outperforming tool, meta-predictor | No website to analyze, for missense only |
| BayesDel | Outperforming tool, meta-predictor | Requires software download to work with |
| GenoCanyon | One of the few unsupervised models | NA |
| Eigen | One of the few unsupervised models, good performance (Li et al, 2018) | NA |
| Nnsplice | One of the first tools available | Old tool without updates, require FASTA input |
| MaxEntScan | Uses entropy | Require FASTA input |
| HSF | Group several splicing algorithm | Paid tool (free credits for academic purposes) |
| dbscSNV | Ensemble splicing tool | No website to analyze, single nucleotide variants only |
| SpliceAI | Newest tool described here based on deep learning | Doesn't support all types of indels |

*NA (Not Applicable): No notable “con” of the tool to expose.
